# Ergonomic Innovation: A Modular Smart Chair for Enhanced Workplace Health and Wellness

**DOI:** 10.3390/s25134024

**Published:** 2025-06-27

**Authors:** Zilvinas Rakauskas, Vytautas Macaitis, Aleksandr Vasjanov, Vaidotas Barzdenas

**Affiliations:** Department of Computer Science and Communications Technologies, Vilnius Gediminas Technical University (VILNIUS TECH), 10105 Vilnius, Lithuania; vytautas.macaitis@vilniustech.lt (V.M.); aleksandr.vasjanov@vilniustech.lt (A.V.); vaidotas.barzdenas@vilniustech.lt (V.B.)

**Keywords:** ergonomic furniture, health monitoring, prolonged sitting, sedentary lifestyle, smart chair, smart technology, wearable devices, workplace ergonomics, wireless communication

## Abstract

The increasing prevalence of sedentary lifestyles poses significant global health challenges, including obesity, diabetes, musculoskeletal disorders, and cardiovascular issues. This paper presents the design and development of a universal smart chair system aimed at mitigating the adverse effects of prolonged sitting. The proposed solution integrates a pressure sensor, vibration motors, an LED strip, and Bluetooth Low-Energy (BLE) communication into a modular and adaptable design. Powered by an STM32WB55CGU6 microcontroller and a rechargeable lithium-ion battery system, the smart chair monitors sitting duration and the user’s posture, and provides alerts through tactile, visual, and auditory notifications. A complementary mobile application allows users to customize sitting time thresholds, monitor activity, and assess battery status. Designed for universal compatibility, the system can be adapted to various chair types. Technical and functional testing demonstrated reliable performance, with the chair operating for over eight workdays on a single charge. The smart chair offers an innovative, cost-effective approach to improving workplace ergonomics and health outcomes, with potential for further enhancements such as posture monitoring. A pilot study with 83 students at VILNIUS TECH showed that the smart chair detected correct posture with 94.78% accuracy, and 97.59% of users responded to alerts by adjusting their posture within an average of 3.27 s.

## 1. Introduction

Globally, 60–85% of people are considered sedentary, both in developed and developing countries. As a result, sedentary behavior has become a serious global health issue, putting them at greater risk of health problems in the future [1].

Prolonged sitting has become a significant and common issue in modern society. Due to technological advancements, computers are now used in nearly every job or field [2]. Doctors, accountants, lawyers, bankers, programmers, taxi and long-haul drivers, and many other modern professions involve long periods of sitting [3]. There are countless scientific publications that have reviewed the risks associated with prolonged sitting [4]. Scientists are studying the effects of prolonged sitting and note that it harms not just the back. They suggest that prolonged sitting can lead to obesity because sitting requires less energy and burns fewer calories. Doctors believe that sitting alters the body’s response to insulin, which is why people with sedentary jobs are at greater risk of developing diabetes [5]. Prolonged sitting can also cause deep vein thrombosis, which may present with leg pain, swelling, or no symptoms at all. A clot formed in the leg can move and travel to the lungs, causing a pulmonary embolism [4].

Finally, prolonged sitting, particularly with poor posture, puts strain on the back, neck muscles, and spine, which can contribute to increased feelings of stress, anxiety, weight gain, and a range of other health issues and conditions [6]. All these risks can be mitigated by taking short breaks from sitting every half hour or hour, exercising, or walking [7].

Another issue related to prolonged sitting at work is particularly noticeable in transportation companies. Scientists and medical professionals emphasize that taxi and long-haul truck drivers face specific health risks, and many in these professions report various problems [8]. According to doctors, common consequences of sedentary work include overweight, elevated cholesterol, and glucose levels in the blood, as well as circulatory disorders [9]. Due to sedentary work and constant vibration, truck drivers also suffer from musculoskeletal disorders, causing back, shoulder, neck, and joint pain. Prolonged muscle tension and vibration can cause structural changes in spinal vertebrae, leading to hernias, which cause pain, hinder movement, and sometimes even result in disability [10]. Additionally, long-haul truck drivers, who spend extended periods on the road and drive without breaks, may lose alertness and focus, which can lead to an increased frequency of accidents and crashes on the road. To reduce the negative impact on health and maintain focus throughout driving, long-haul drivers are allowed to drive no more than 9 h per day (with up to 10 h permitted twice a week), with a mandatory 45-min break after 4.5 h of driving [11].

To address these problems and meet health requirements, it is essential to create smart devices that monitor and collect information about sitting duration and notify the person or their employer about prolonged sitting during work hours. For these reasons, it is necessary to develop various smart sitting monitoring systems that can track how long a person sits and the quality of their sitting posture and notify the chair’s user about it.

This article introduces a novel smart chair system designed to mitigate the health risks associated with prolonged sitting. Unlike existing solutions that focus on individual components, this system integrates pressure sensors, vibration motors, an LED strip, and BLE communication into a modular and universally adaptable design. Its comprehensive approach, including real-time feedback via a mobile app, distinguishes it from previous products, offering a practical and innovative solution to improve workplace ergonomics and health outcomes.

This paper is organized as follows: the introduction is followed by a review of analogous systems. The next sections present the development of the proposed smart chair in detail, including the block diagram, sketch, and hardware and software design. Conclusions and references are provided at the end of the paper.

## 2. Review of Analogous Systems

After a detailed analysis of products on the market, only a few attempts at creating smart chairs were observed, but there have been no final, successful commercial products.

One example of such a chair could be the Gregory Smart Chair, developed by Australian company Gregory Chairs, which is a system compatible with their chairs that tracks sitting duration, posture, and connects to a mobile app via Bluetooth LE. It alerts users about prolonged sitting or poor posture, helping improve health and productivity by reducing back issues and deep vein thrombosis risks. The free mobile app, available for Android and iOS, allows users to monitor sitting habits, adjust break settings, access chair adjustment guides, request quotes, track warranties, and submit repair requests. The chair connects to the app via a QR code, and the interface displays sitting times, daily totals, and break statistics [12].

The second example of an AI Smart Sitting Module (AI ASM) could be the Swingsit company’s product—an active sitting chair. This chair stands out for its functionality. It integrates electronics that give it the ability to combat the consequences of prolonged sitting. The principle of the chair is to move or sway side to side while sitting. According to the manufacturer, this system combines walking and sitting in one, so while sitting for long hours, the person is also exercising, burning calories, improving posture, and enhancing blood circulation. In this chair, only the seat moves constantly in both directions. While the chair moves, the person’s head and shoulders remain stationary, and only the waistline moves, providing physical activity at the same time. For these reasons, this chair is more suited to work rather than rest or relaxation. However, research has shown that these chairs reduce a person’s concentration when working on complex, focused tasks. This smart chair was first sold in 2019 but is no longer available due to a lack of popularity [13].

The third example of an AI ASM could be the “Routine Chair” project, created by designers Yubin Lee and Minkyoung Song. This chair is unique in that the tilt of the seat and armrests can be controlled via a mobile device app. The positions of the backrests can be set for various modes, for instance, making the chair more reclined or upright at certain times. The chair can also be configured for different activities—working, taking a break, or resting. However, this chair has not been physically implemented and is presented to the public only as a vision with potential implementation methods [14].

There are quite a few scientific articles that have conducted research on smart furniture, such as wheelchairs, aimed at enhancing mobility and safety for individuals with paralysis. For example, a previous article [15] introduced an IoT-enabled wheelchair equipped with various sensors, autonomous navigation, and intuitive controls such as voice and touch interfaces, where data processing takes place in real time or through a cloud connection. Another article [16] presented a novel electronic wheelchair design that integrates AI-assisted smart sensors and controllers to enhance functionality and user experience. It features environmental and health monitoring through sensors like ECG, oxygen, and heart rate, while an AI-driven controller optimizes navigation. All these smart wheelchairs being developed have very extensive and complex functionality, which is not necessary when designing ordinary office chairs intended for monitoring sitting duration and posture. This would allow for the simplification of the electronics and a reduction in the cost of such a smart chair.

The scientific literature also presents numerous attempts to develop smart chairs with complex designs, incorporating a wide range of pressure and image sensors to monitor various sitting postures and determine whether the user is seated correctly [17,18]. However, such solutions are more closely associated with futuristic technologies, potentially intended for the development of next-generation office or household furniture. In contrast, with our smart chair design, we aim to demonstrate that by integrating smart seat and backrest cushions along with a user-friendly mobile application, it is possible to adapt any existing office or household chair for effective sitting duration and posture monitoring.

There have also been scientific studies that have attempted to develop smart chairs similar to the one presented in this work. One such attempt is described in another article [19], where the authors propose a seat cushion placed on a chair to monitor a person’s sitting behavior. However, in our development of a smart chair, we have also implemented backrest monitoring functionality to provide a more comprehensive assessment of proper sitting posture. Additionally, we introduced a smartphone application feature that allows users to track their sitting duration and posture correctness.

While there are many traditional chairs on the market that prioritize ergonomics, softness, or comfort, none currently feature integrated smart electronic systems that monitor sitting duration and posture or provide reminders to encourage walking or physical activity. The absence of such technology leaves a significant gap in addressing the health risks associated with prolonged sitting, including obesity, musculoskeletal disorders, and cardiovascular issues. The proposed smart chair system fills this gap by offering a comprehensive solution that can be seamlessly integrated into various environments such as homes, workplaces, cars, or during travel. By continuously monitoring sitting behavior and providing real-time feedback through tactile, visual, and auditory cues, the device promotes healthier habits, ensuring users remain active and mitigate the adverse effects of sedentary lifestyles.

## 3. Smart Chair Model Development

### 3.1. Overall System Design

The smart chair is designed with a modular and configurable architecture that enhances workplace ergonomics via real-time monitoring and feedback. On a high level, the system consists of two smart cushions with embedded pressure sensors and vibration motors, a control unit that houses the microcontroller and power supply, an LED strip for visual alerts, and a mobile app for user interaction. The chair detects whether a person is seated and monitors posture using pressure sensors in the seat and backrest. When the system identifies prolonged sitting or poor posture, it provides visual, tactile, or audio signals to prompt the user to change their behavior. The STM32WB55CGU6 microcontroller was chosen for its integrated Bluetooth Low-Energy (BLE) capabilities, which enable easy connectivity with the mobile app. The sensors, which are adapted from automobile seat occupancy systems, provide accurate and consistent readings, while small vibration motors provide slight but discernible input. Powered by rechargeable lithium-ion batteries and governed by efficient electronics, the system allows for extended operation without regular charging, making it a practical and user-friendly option. The functions of the sensors and components mentioned above are summarized in Table 1.

Table 1 describes how each sensor and component contributes to the smart chair’s overall functionality. This integration enables the system to efficiently monitor user activity and deliver timely feedback to promote better seating patterns.

While conceptualizing and developing the idea for a smart chair, various versions were considered on how to integrate the electronic system with the chair into a single final product. It was decided to proceed with a version of the smart chair system designed to allow easy adaptation to any chair type, whether it be an office chair, an executive chair, or a simple chair without wheels. The main advantage of this smart chair system is its universality. Given the wide variety of chairs differing in color, material, quality, and price, the smart chair system can be adapted to virtually any chair. It is recommended to use a chair with a backrest to simplify the installation of the LED strip.

The final design of the smart chair was chosen with universality in mind. The smart chair consists of the following components: the chair, smart pillows, control unit, and LED strip.

The smart pillow, which contains an integrated pressure sensor (red color) and vibrating elements (green color), is attached to the seating area of the chair. The control unit, which includes the PCB, two lithium-ion batteries, and a sound alarm, is mounted under the seating area. The LED strip is affixed to the outer side of the chair’s backrest. Since the LED strip is mounted externally, the user will not see it while seated. Its primary function is to inform the user whether the chair is occupied, whether it is safe to sit, and approximately how much time is left before they need to stand up, if required.

**Figure 1:**
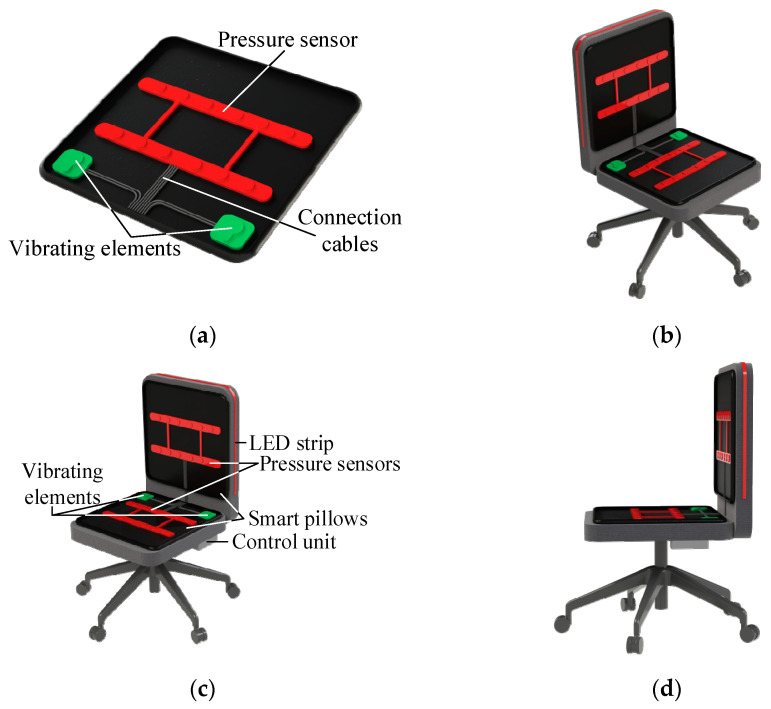
Smart chair’s construction design sketch: (**a**) smart pillow design; (**b**) full smart chair perspective; (**c**) labeled smart chair; (**d**) side-view smart chair perspective.

Preliminary final sketches of the smart chair are illustrated in Figure 1.

The provided illustration demonstrates the structural design and integration of components within the smart chair system from multiple perspectives.

### 3.2. Block Diagram of the Smart Chair’s Electronic System

**Figure 2:**
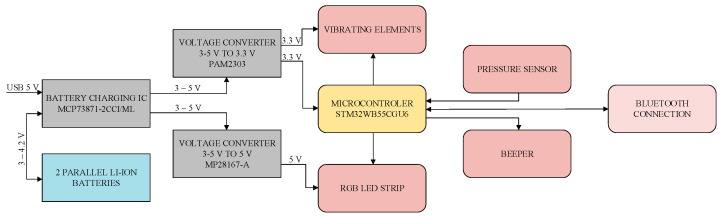
Block diagram of the smart chair’s electronic system.

When designing the structural diagram, it is essential to consider the functions of the electronic part of the smart chair to be implemented. A block diagram of the smart chair’s electronic part was created, as shown in Figure 2. The block diagram was designed using the Draw.io 24.0.4 software package.

In Figure 2, the gray elements represent power ICs, orange represents the microcontroller, pink represents peripherals, and light pink represents wireless communication. The first gray element is the MCP73871-2CCI/ML battery charging IC, which is connected to a USB 5 V input indicated by an arrow. This IC powers the circuit with 5 V and charges the batteries if connected. A bi-directional arrow connects the IC to two parallel lithium-ion batteries with 3–4.2 V output. The bi-directional arrow indicates that when a 5 V input is present, the IC charges the batteries, and when the 5 V input is absent, the IC draws power from the batteries at 3–4.2 V, depending on their charge level. The IC outputs two voltages (3–5 V) to the 3.3 V and 5 V converters. The 3.3 V output powers the microcontroller and vibration elements, while the 5 V output powers the LED strip. The powered microcontroller connects to the LED strip, acoustic signaler, pressure sensor, and vibration elements. Wireless communication is linked bidirectionally to the microcontroller, as the BLE module sends and receives data.

The proposed smart chair is powered by an autonomous power source; thus, two 18,650 lithium-ion batteries with a capacity of 2500 mAh were selected [6]. This decision was influenced by the expected size of the printed circuit board (PCB), the possibility of accommodating more than one battery within the casing, the possibility of easily changing or charging the batteries, their power density and longevity, and the availability of the latter type of batteries.

An STM32WB55CGU6 microcontroller from the STM32 family was chosen due to its integrated Bluetooth Low-Energy (BLE) interface.

A car seat pressure sensor was selected to detect that a person is sitting, as these sensors are readily available, robust, and commonly found in car parts stores. Their flat form factor makes them easy to integrate into the smart chair, and they operate simply: when pressure is applied at several points of the sensor, a circuit with a resistance of a few hundred ohms is formed; when no pressure is applied, the sensor acts as an open circuit. However, a limitation of this sensor is that regardless of the number of points under pressure, the circuit’s resistance remains constant, making it impossible to determine the sitting posture.

To inform the user about prolonged sitting, a tri-color LED strip, an acoustic signaler, and two vibration elements were selected. These components provide various types of feedback, signaling different stages of prolonged sitting. For instance, they notify the user when more than half of the permitted sitting time remains, when the time is nearly up, or when excessive sitting has occurred.

A USB-C connector was chosen for charging the batteries with a 5 V power source, such as a computer or external power adapter, as well as programming the microcontroller. This connector allows connecting to the microcontroller without additional programmers. Additionally, the microcontroller can still be programmed via a Serial Wire interface, but this requires a dedicated ST-LINK programmer. The USB-C interface was selected for its convenience—allowing cables to be plugged in either way—and its widespread use in electronic devices, as USB-C cables are readily available to users. Furthermore, USB-C has become almost a universal standard for connectivity, ensuring broad compatibility with a wide range of devices.

According to the microcontroller’s datasheet and the peripherals used, it was determined that the system requires a 3.3 V power circuit for the microcontroller and vibration elements and a 5 V power circuit for the LED strip. Other peripherals are powered and controlled by the microcontroller. Additionally, a battery charging integrated circuit (IC) is required to charge the lithium-ion batteries, control the charging current and voltage, protect the circuit from reverse battery polarity, stop charging at high temperatures, etc. Since the required input voltage is up to 5 V, two lithium-ion batteries are connected in parallel. This configuration increases the total battery capacity while maintaining the same voltage.

The prototype was created as a universal modular kit that can be seamlessly integrated into a wide range of chair types, including workplace swivel chairs, vehicle seats, and plain wooden chairs. The core components (smart cushions with pressure sensors and vibration motors, the microcontroller-based control unit, and the LED strip) attach without structural modifications: the cushions rest on the seat surface, the LED strip mounts externally on the backrest (invisible to seated users), and the control unit with lithium-ion batteries is secured beneath the seat (Figure 1). This modular architecture allows for simple installation and mobility between chair types, retaining complete functionality.

The system lasted over 8 workdays (64 h) per charge, due to the energy-efficient STM32WB55CGU6 microprocessor and dual-battery management.

The system’s key strength is proactive health promotion. Multimodal signals (vibration, light, and sound) reduce the risk of prolonged sitting, while the mobile app allows for personalized thresholds and alarm customization. Automotive-grade pressure sensors provided long-lasting performance.

## 4. Chair Hardware and Software Design

The hardware and software design of the smart chair is the most critical stage in the development of this product. The electronics design for the smart chair includes the hardware aspects, such as drawing the block diagram (Figure 1), creating the schematic diagram, selecting the microcontroller, designing the PCB, designing the PCB housing, and constructing the entire system. However, the design process also involves the software aspect, which includes creating the program algorithm and programming the microcontroller, as well as the smart device application.

The schematic diagram and PCB design were created using the Altium Designer 25.3 software package. This comprehensive approach ensures both the hardware and software components work seamlessly together, resulting in a fully functional smart chair system.

### 4.1. Development of the Smart Chair Electric Diagram

The primary circuit components for the smart chair’s electronics are outlined below.

The USB-C interface provides power and data connectivity. It uses filtering components like capacitors and ferrite beads to stabilize signals and includes protection features such as ESD diodes to safeguard against electrostatic discharge.

The battery charging circuit, implemented with the MCP73871-2CCI/ML, manages charging for lithium-ion batteries. It can charge while supplying power to the circuit, includes protection against reverse polarity, and allows adjustable charging rates for efficient thermal management.

Regarding power supply circuits, the 3.3 V voltage converter, based on PAM2303, steps down battery voltage to 3.3 V for low-power components. It features a stable output regulated by feedback resistors and capacitors.

The 5 V voltage converter, built around MP28167-A, boosts voltage to 5 V for components requiring higher power. It includes filtering elements to minimize noise and ensure stable output for reliable operation.

The UART interface circuit, utilizing FT230XQ-R, facilitates serial communication via USB for programming the microcontroller. It also supports fast charging by signaling the battery charging IC.

The microcontroller circuit, centered on STM32WB55CGU6, integrates functionalities like Bluetooth communication, ADC for battery voltage monitoring, and GPIOs for controlling peripherals such as LEDs, vibrators, and sensors.

The Bluetooth interface handles wireless communication, enabling remote control and data exchange with external devices. It includes impedance matching and filtering to ensure stable RF performance.

Additional components include pressure sensors, vibration motors, LED strips, and various filtering and protection elements, ensuring reliable performance and efficient power management throughout the system.

### 4.2. Program Algorithm Design

**Figure 3:**
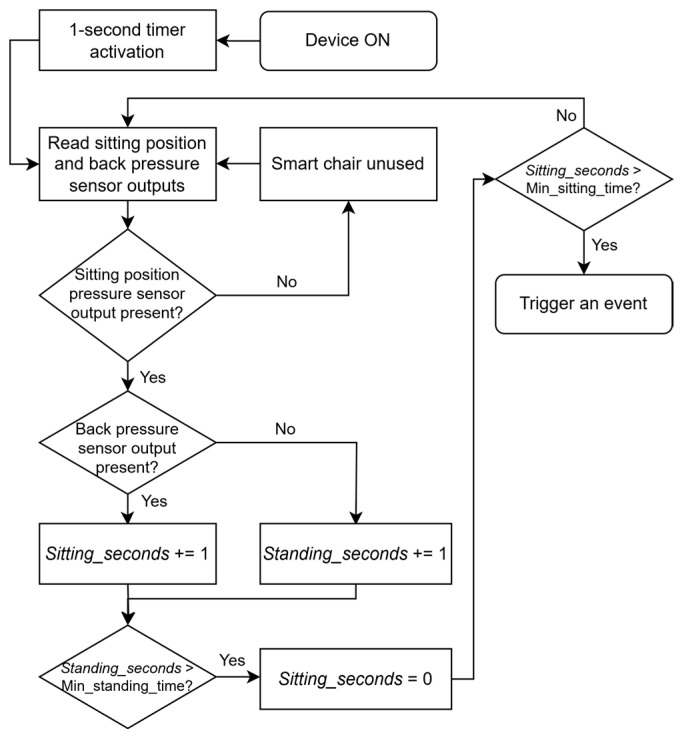
Algorithm of microcontroller seat tracking program.

Creating a program algorithm before starting programming is beneficial as it can save time during code writing. It becomes easier to develop software when an algorithm is pre-planned, outlining how the program should operate and how the device should function. The microcontroller sitting detection program algorithm is depicted in Figure 3.

The microcontroller seat-tracking program begins operation when the device is powered on. A consistent one-second timer activates to maintain regular sensor sampling intervals throughout the program’s execution cycle.

During each cycle, the system reads data from two key sensors: the sitting position pressure sensor and the back pressure sensor. The program first evaluates whether the sitting position pressure sensor detects any output. If no pressure is detected on the seat, the system recognizes that the smart chair is currently unused.

However, if sitting pressure is detected, the algorithm proceeds to check the back pressure sensor output. When back pressure is present, indicating proper seated posture with back support, the program increments a “*Sitting_seconds*” counter to track duration in this position. Alternatively, if no back pressure is detected despite seat occupation, the system increments a “*Standing_seconds*” counter and simultaneously resets the sitting counter to zero.

The program Incorporates two Important time thresholds. If the “*Standing_seconds*” counter exceeds a predetermined minimum standing time, the system triggers an event. Similarly, if the “*Sitting_seconds*” counter surpasses a minimum sitting time threshold, an event is also triggered.

After completing each evaluation cycle, the program loops back to the timer activation step, continuing the monitoring process. This creates a continuous feedback system that effectively tracks sitting behavior through sensor data, allowing for appropriate responses based on timed thresholds of different posture states.

**Figure 4:**
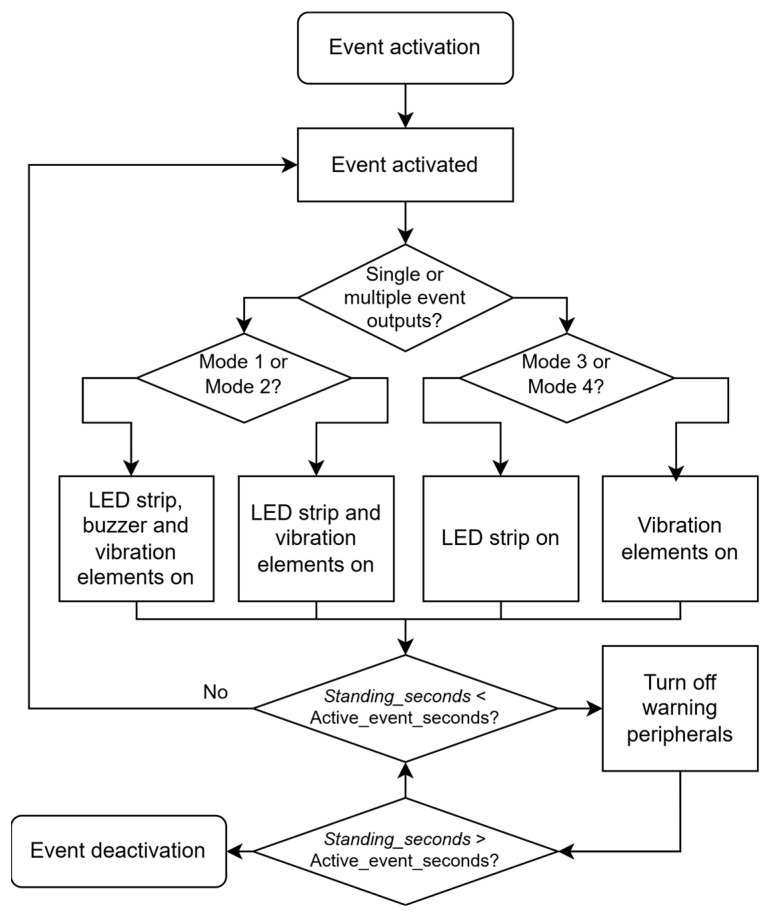
Algorithm of sitting event.

The sitting event algorithm is shown in Figure 4. The sitting event algorithm begins with event activation, which occurs when specific time-based conditions are met in the seat tracking system. Once activated, the algorithm determines whether to produce single or multiple event outputs.

Based on this decision, the system proceeds through different operational modes. If Mode 1 or Mode 2 is selected, the algorithm activates specific combinations of warning peripherals. In Mode 1, the system turns on the LED strip, buzzer, and vibration elements simultaneously to provide maximum notification. In Mode 2, a more subtle approach is taken, with only the LED strip and vibration elements being activated.

Alternatively, if Mode 3 or Mode 4 is selected, different peripheral combinations are utilized. Mode 3 only activates the LED strip, providing a visual cue without tactile or audio feedback. Mode 4 only enables the vibration elements, offering discrete tactile notification without visual indicators.

After activating the appropriate notification peripherals, the system continuously monitors the “*Standing_seconds*” counter. The algorithm checks whether this value remains below a predetermined “*Active_event_seconds*” threshold. If this condition persists, the notifications remain active.

However, once the “*Standing_seconds*” counter exceeds the “*Active_event_seconds*” threshold, indicating sufficient time spent in a standing position, the algorithm initiates the deactivation process. This involves turning off all active warning peripherals and completing the event deactivation sequence.

The algorithm is designed to encourage healthy sitting habits by providing configurable notifications when sitting time thresholds are exceeded and automatically ceasing these notifications once the user has stood for an adequate duration.

Although these algorithms operate sequentially, the high 64 MHz clock frequency of the microcontroller ensures commands are executed rapidly, giving the impression of parallel operation. This algorithm provides flexibility for user-defined settings, improves energy efficiency, and ensures a better user experience by separating active and passive event states.

### 4.3. Printed Circuit Board (PCB) Design

The design of the smart chair system’s PCB started with selecting the layer configuration. An affordable 4-layer PCB was chosen because it provides additional design flexibility and is well-suited for high-frequency (RF) line design. Specifically, having a continuous ground plane directly beneath the RF line is highly recommended to ensure signal integrity and minimize interference.

The layer thicknesses and dielectric material were selected based on recommendations from the PCB manufacturer JLCPCB, as it was planned to order the PCB from them. These specifications ensure compatibility with the manufacturing process and optimal performance for the system.

**Figure 5:**
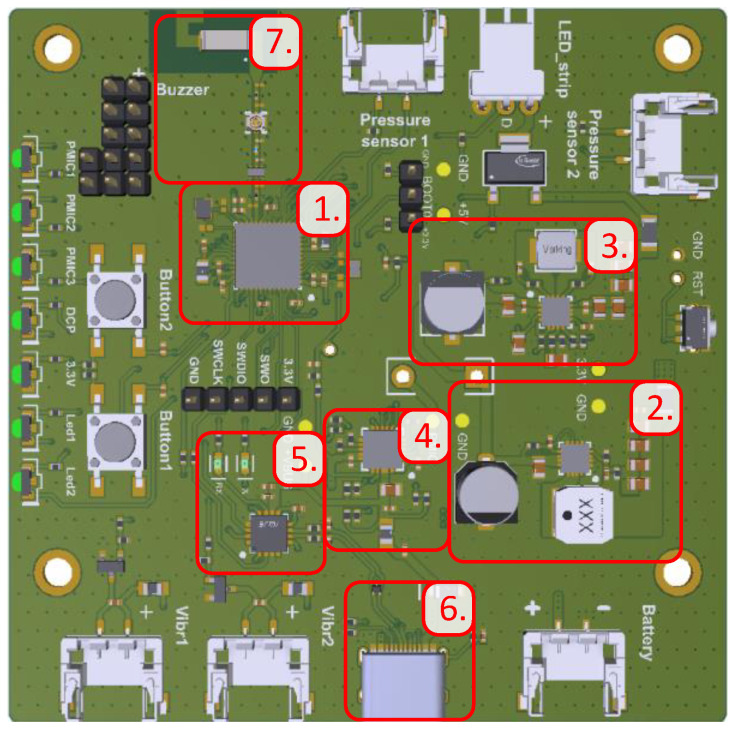
The designed PCB (numbers outlined in red are described in detail in the text).

The final PCB design is presented in Figure 5. The PCB design for the smart chair system incorporates several important features and considerations:

The microcontroller and associated components are placed to optimize space, ensuring shorter traces and close placement of capacitors to the IC for stability (1) (numbers outlined in red shown in Figure 5). The 3.3 V (2) and 5 V (3) voltage converters were designed based on datasheet recommendations, with wide power traces to reduce resistance and improve heat dissipation. The 5 V converter, being more complex, includes additional resistors for its operation.

The battery charging circuit was designed with the compact placement of resistors used in voltage dividers, prioritizing proximity to the IC for efficient use of space (4). For the FT230XQ-R interface, attention was given to the USB data lines, which are routed as a 90 Ω differential pair, and power traces are widened to handle higher currents without overheating (5).

The USB-C connector includes a 0.7 mm power trace that broadens to 1 mm to support sufficient current flow (6). The RF line was designed to be as short as possible, with a ground shield to minimize reflections and interference, ensuring reliable BLE communication (7).

Peripherals such as indicator LEDs, buttons, and connectors were strategically placed for ease of use during testing and in the final enclosure. All LEDs were grouped on one edge for better visibility. The PCB layout also includes mounting holes, test points, and silk screen labels for peripheral connectors, BOOT0 pins, and LEDs, simplifying its use and testing.

### 4.4. Construction of the Device Prototype and Mobile Application

**Figure 6:**
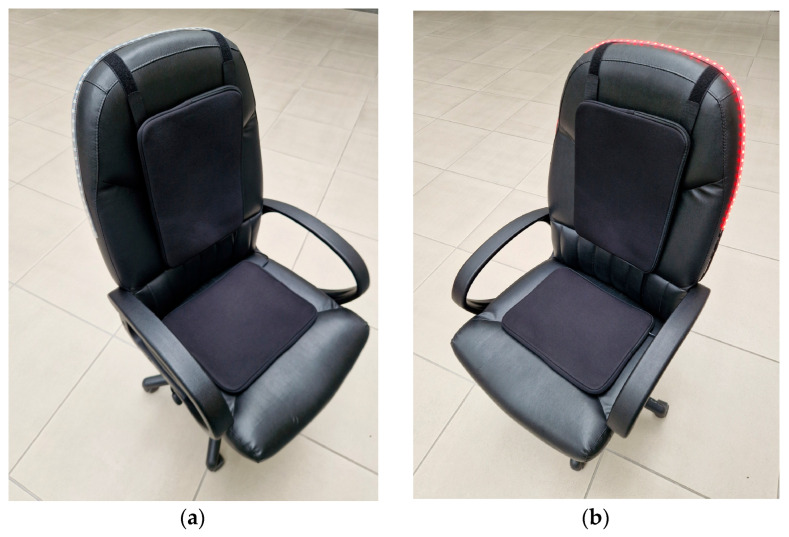

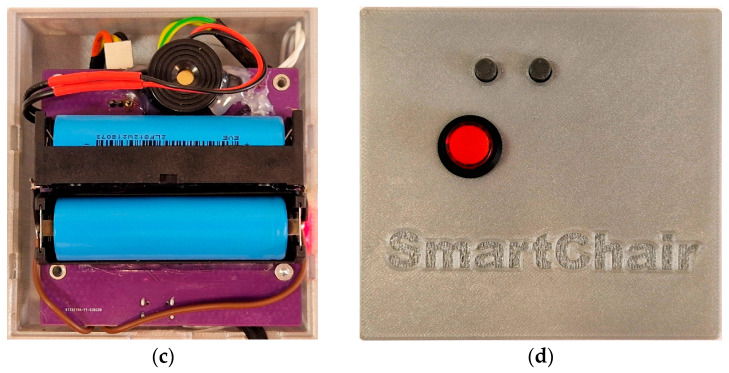
Smart chair system: (**a**) a full view of the smart chair with the integrated system; (**b**) a view of the smart chair with the LED strip activated; (**c**) the internal electronics of the smart chair control unit; and (**d**) the external casing of the smart chair’s control unit.

**Figure 7:**
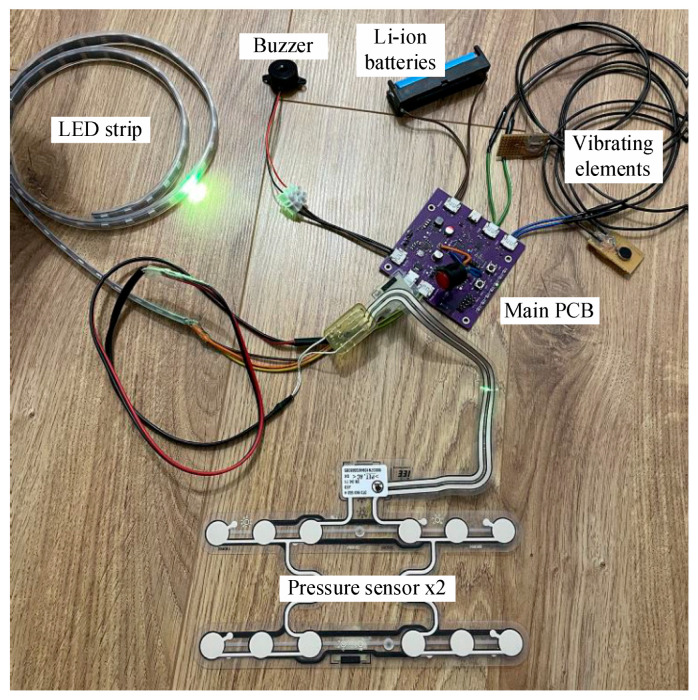
The main electronic components of the smart chair system.

The entire constructed system is shown in Figure 6 and Figure 7. The system consists of several interconnected components integrated into the smart chair.

Figure 6a shows the smart chair in its assembled form, featuring a standard chair with a cushion on the seat. The pillow houses the integrated pressure sensor, used to detect when someone is sitting, and vibrating components. This view presents the smart chair in its default state, without any active alerts or visible indicators.

Figure 6b shows the smart chair in action, with the LED strip on the backrest illuminated in red. This indicates that the system has detected prolonged sitting and activated a visual alert. The LED strip serves as a warning mechanism, signaling the user that it is time to take a break or stand up. The integration of the LED strip into the backrest ensures that the alert is visible while maintaining the chair’s overall aesthetic and functionality. This active state demonstrates how the smart chair provides feedback in response to user behavior.

Figure 6c provides an internal view of the smart chair’s control unit. It shows the electronics housed within the enclosure, including the PCB, two lithium-ion batteries, and associated wiring. The PCB contains the microcontroller and other key components that power and control the system. The layout is compact, ensuring all parts fit securely within the enclosure. The design allows for the efficient connection of peripherals such as the pressure sensor, LED strip, buzzer, and vibration motors while maintaining easy access for assembly or maintenance.

The enclosure for the smart chair’s microcontroller-based device was designed using the Fusion 360 software 2024. The enclosure created was larger than the PCB to accommodate not only the PCB itself but also two lithium-ion batteries, a buzzer, and sufficient space for the warning peripherals’ wires and connectors. Considering these requirements, the device’s enclosure, shown in Figure 6d, was designed with the dimensions 101.1 mm in length, 94.5 mm in width, and 51 mm in height.

The main electronic components of the smart chair system are shown In Figure 7. The LED strip provides visual feedback and notifications. The buzzer generates audible alerts or notifications. The Li-ion batteries serve as the primary power source for the system. The main PCB integrates the circuit design, including the microcontroller and power management components. The vibrating elements provide feedback for user interaction. The pressure sensor detects the user’s presence and monitors sitting activity.

This coin-shaped flat vibration motor operates on DC 3 V with a current of 60 mA and delivers a rotational speed of 9000–12,000 RPM. Despite its compact size, it produces a distinct and noticeable vibration, making it ideal for mobile phones and similar devices. The motor features two lead connections for easy integration into electronic circuits, offering reliable tactile feedback in a space-efficient form factor [20].

The automotive seat pressure sensor utilizes resistance changes to detect occupancy, operating at 12 V with minimal current draw (<10 mA standby, 20–50 mA active). Resistance ranges from 300–600 Ω when unoccupied to 50–150 Ω with an adult occupant. The sensor demonstrates exceptional reliability and longevity, as required for automotive systems, enabling consistent weight-based detection for critical vehicle safety functions [21].

**Figure 8:**
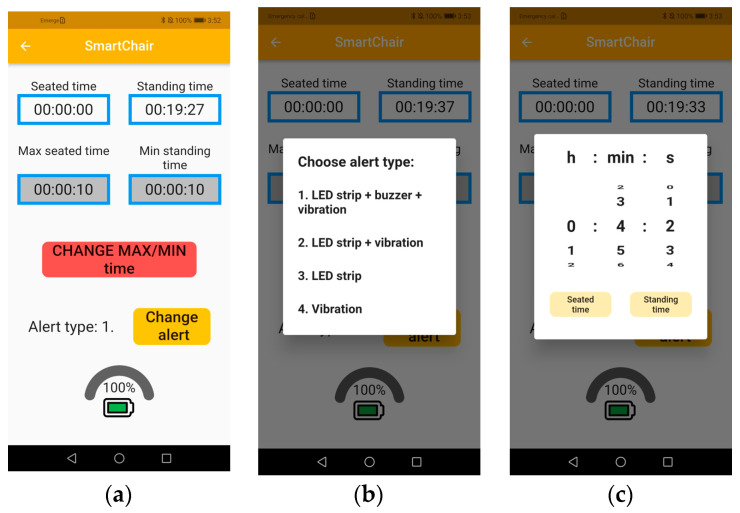
Smart chair mobile application interface: (**a**) main interface window; (**b**) pop-up window for selecting and setting the alert type; (**c**) pop-up window for modifying the maximum seated time and minimum standing time.

A dedicated mobile application was developed for Android devices to control and monitor the smart chair system. Figure 8 illustrates the interface visible when the application connects to the Smart Chair device. The screen displays the seated time, standing time, maximum seated time, minimum standing time, and currently set alert type.

In Figure 8a, the user interface shows all these values in a clean layout, allowing the user to monitor their activity. By pressing the red button labeled “CHANGE MAX/MIN time”, a window appears (as shown in Figure 8c), enabling the user to modify the maximum seated time and minimum standing time. Pressing the yellow “Change alert” button opens another window (as shown in Figure 8b), where the user can select and set one of the available alert types. The alert types include combinations of the LED strip, buzzer, and vibration elements, allowing customization based on user preferences.

This application provides an intuitive way to interact with the smart chair, making it easier for users to track and adjust their sitting behavior.

## 5. Smart Chair Testing

### 5.1. Testing

After conducting functional tests of the electronic device, it was determined that the microcontroller program operates correctly. The battery voltage was measured with minimal error compared to measurements taken with a multimeter. The system accurately detected sitting and standing, and the timing generated by the microcontroller had a deviation of 3 s per 5 min. However, this deviation was negligible. The maximum sitting time and minimum standing time can be adjusted correctly.

Testing of the mobile application confirmed that it functions well. The app successfully detects and connects to the Smart Chair every time it is powered on. Even if the app is closed improperly, reopening it allows for successful reconnection to the Smart Chair without issues.

This research tested a newly developed smart chair equipped with pressure sensors on the seat and backrest, vibration elements, and an LED warning system designed to monitor correct sitting posture. The testing took place in the laboratory of VILNIUS TECH, Faculty of Electronics, involving all 83 students from the faculty.

Each student was required to sit on the smart chair three times for 3 min during the test. A 1 min break was taken between each trial. All participants had their height and weight measured before testing. The weight of students in the test group ranged from 60 kg to 100 kg, and height from 160 cm to 193 cm.

During testing, the chair monitored whether the student was sitting correctly according to pre-established parameters. If the posture became incorrect, the chair activated vibration elements and an LED warning. Throughout the testing, data was recorded for each participant: height, weight, duration of correct/incorrect posture, and accuracy of the chair’s response.

The student distribution by weight and height is presented in Table 2 and Table 3. The student group was dominated by individuals with heights of 171–180 cm, comprising 42.17% of all participants. In the weight categories, the largest proportions were 60–70 kg (39.76%) and 71–80 kg (38.55%) students. Of all 83 participants, only 3 students (3.62%) belonged to the highest weight category (91–100 kg).

The testing was conducted over four days, during which all 83 students participated in the study. First, students were introduced to the operating principles of the smart chair, and the testing goals and procedure were explained. All participants were assigned ID numbers, and their demographic and anthropometric data were recorded in the pre-testing phase.

Each student began testing by sitting on the chair and assuming a comfortable position. After a signal, students were instructed to sit naturally while performing typical computer work activities. The first 3-min phase was dedicated to observing natural sitting posture. During the second trial, students were asked to consciously maintain correct posture. In the third trial, students were instructed to alternate between correct and incorrect posture every 30 s.

During testing, the observers recorded not only the chair’s performance but also students’ reactions to vibration and LED warnings. The time taken for students to correct their posture after receiving a warning was also documented.

The results showed high effectiveness of the smart chair in detecting incorrect posture. The system performed particularly well with students of average weight and height. It was observed that the chair less frequently identified incorrect posture for lighter (60–70 kg) and shorter (160–170 cm) students.

An analysis of the test results is shown in Table 4. According to this table, out of the 83 students, 97.59% corrected their posture after receiving a signal from the chair, indicating high system effectiveness. The average response time to a warning was 3.27 s, and the overall accuracy of correct posture recognition was 94.78%.

The results according to students’ anthropometric data are shown in Table 5. Interestingly, the smart chair performed most accurately with students of medium and higher weight. The highest detection accuracy (98.75%) was achieved with students weighing 91–100 kg and measuring 18–193 cm in height. Meanwhile, in the group of shorter (160–170 cm) and lighter (60–70 kg) students, the accuracy was 88.42%, indicating that the system has more difficulty detecting incorrect posture when less pressure is applied to the sensors.

### 5.2. Discussion of Results

The observed differences in posture identification accuracy—ranging from 88.42% for lightweight users (60–70 kg) to 98.75% for heavier users (81–100 kg)—are mostly related to sensor physics constraints and static system architecture. Automotive-grade pressure sensors require a minimum force threshold (~50–100 Newtons) to detect occupancy, resulting in inconsistent triggering for lighter users who exert lower pressure, especially on cushioned surfaces. This difficulty is compounded by unequal weight distribution in uncommon postures (for example, cross-legged sitting), where targeted pressure points entirely miss sensor zones. Fixed-position sensors may misalign with spinal contact points, while deep cushion foam reduces pressure transfer. The binary identification system does not detect slow posture alterations, and the lack of a user profile ignores physiological differences, resulting in underfitting of outlier physiques.

To overcome these limits, a diversified approach is required. To map spatial pressure distribution, hardware should contain high-sensitivity piezoresistive sensor grids, rather than single-point sensors. Cushion foam can be changed with variable density layers-softer tops for pressure concentration and firmer bases for signal amplification to improve sensitivity for lightweight users. Complementary proximity sensors (infrared) on the backrests would detect changes in spine distance independent of pressure. Dynamic threshold calibration should be employed instead of static logic: a 30 s initial setup would provide tailored baselines by adjusting thresholds for sitting length and posture variation. Mechanical advancements, such as repositionable sensor pods and lumbar-specific cushion cavities, will keep the spine in proper alignment.

These advantages would be bolstered by user-friendly software features. A mobile app calibration mode might instruct users to “press firmly on the backrest for 5 s” to establish unique baselines, yet multi-profile support allows shared chairs.

While these enhancements would considerably improve accuracy and user inclusivity, they would also increase complexity and cost. Higher-quality sensors, mechanical modifications, and software development would increase the chair’s production costs. This presents a dilemma because our original goal was to create a product that was broadly applicable and socially accessible—something inexpensive, simple to use, and generally adopted. As we explore the next steps, we must continue to balance technical performance with price.

While these enhancements would improve accuracy, they would also increase system complexity and production costs. Higher-quality sensors, mechanical enhancements, and additional software features would raise the chair’s overall cost. This contradicts the original goal of creating a widely relevant and socially accessible product that is inexpensive, easy to use, and well-suited for widespread adoption. Balancing technical performance and affordability will remain a fundamental design challenge in the future.

## 6. Conclusions

In this work, a universal smart chair is presented, designed to address health problems associated with prolonged sitting. The chair’s purpose is to monitor the user’s posture and sitting duration, encourage more frequent breaks, and help maintain correct posture. The system integrates pressure sensors in the seat and backrest surfaces, vibrating elements, an LED strip, and an audio signal. The entire system is controlled by an STM32WB55CGU6 microcontroller, which operates with two rechargeable lithium-ion batteries and communicates via Bluetooth Low Energy with a mobile application. Through the app, users can monitor sitting duration, change the type and frequency of alerts, check the battery level, and adjust settings according to their needs.

A major advantage of this chair is its versatility. It is not dependent on a specific chair design: the main components, such as the smart cushions, control unit, and LED strip, can be integrated into various chair types: office, work, leisure, or even car seats. The chair responds to the user’s posture: if a person sits incorrectly or for too long, visual, audio, and/or vibration alerts are activated. The system also automatically calculates how long the user has been sitting or standing, displays information on the mobile device accordingly, and sends signals when the set sitting time is exceeded or when posture is incorrect.

To evaluate the prototype’s performance, a pilot study was conducted at the VILNIUS TECH Electronics Faculty laboratory. The study involved 83 students who sat on the smart chair three times for three minutes each: naturally, with correct posture, and alternating postures. The system monitored posture changes and activated appropriate alerts. The results showed that the chair recognized the correct posture with 94.78% accuracy, and 97.59% of participants responded to alerts and corrected their posture in an average of 3.27 s. It was observed that the system worked most accurately with medium- and larger-built participants, while sensitivity decreased slightly with lower-weight participants. From this study, one can conclude that the chair functions correctly.

This smart chair or its components could be applied in various contexts. In home environments, it can help elderly people or those in rehabilitation maintain activity and correct posture. In offices, it is beneficial for employees who spend a lot of time at computers, helping prevent spine and neck problems and reducing tension. In the transportation sector, especially on long-distance routes, integrated chair cushions could help maintain alertness and remind them about necessary breaks. Also, in educational institutions and universities, such a solution would promote healthier work and learning habits. Using such technology can reduce the risk of chronic health disorders, musculoskeletal diseases, and circulatory problems.

In the future, the system could be further improved. Increasing the sensitivity and number of sensors would make posture recognition even more accurate, regardless of the user’s body composition. In the mobile application, it would be useful to integrate long-term statistics tracking, allowing for an analysis of habits and progress monitoring. Also, an important aspect is energy consumption optimization, aiming for even longer operation time without recharging.

## Figures and Tables

**Table 1:** Functions of sensors and components.

Function	Sensor/Component Used	Description
Sitting detection	Seat pressure sensor (automotive)	Detects user presence via applied pressure.
Posture monitoring	Backrest pressure sensor	Assesses posture correctness based on back support contact.
Tactile alert	Coin-style vibration motors	Delivers physical prompts to adjust posture or take breaks.
Visual alert	Tri-color LED strip	Indicates occupancy, time thresholds, or warnings.
Auditory alert	Buzzer	Emits sounds for critical posture/sitting duration violations.
Data processing	STM32WB55CGU6 microcontroller	Manages sensor inputs, alert logic, and BLE communication.
Power supply	Dual 18,650 Li-ion batteries	Supports > 8 workdays of operation per charge.
Wireless communication	BLE module + mobile app	Enables real-time data transfer and user customization.

**Table 2:** Student distribution by weight.

Weight Category	Number of Students	Percentage
60–70 kg	33	39.76%
71–80 kg	32	38.55%
81–90 kg	15	18.07%
91–100 kg	3	3.62%

**Table 3:** Student distribution by height.

Height Category	Number of Students	Percentage
160–170 cm	28	33.73%
171–180 cm	35	42.17%
181–193 cm	20	24.10%

**Table 4:** Summarized testing results.

Parameter	Result
Overall accuracy of correct posture recognition	94.78%
Sensitivity of incorrect posture detection	91.23%
Frequency of false warnings	5.42%
Average response time to warning	3.27 s
Percentage of students who corrected posture after warning	97.59%
Break reminder effectiveness (3-min intervals)	100.00%

**Table 5:** Results according to students’ anthropometric data.

Weight (kg)	Height (cm)	Detection Accuracy (%)	Number of Students
60–70	160–170	88.42%	15
60–70	171–180	90.17%	12
60–70	181–193	91.56%	6
71–80	160–170	92.84%	9
71–80	171–180	95.93%	15
71–80	181–193	96.12%	8
81–90	160–170	93.27%	4
81–90	171–180	97.41%	6
81–90	181–193	98.05%	5
91–100	171–180	97.89%	2
91–100	181–193	98.75%	1

## Data Availability

Data is contained within the article.

## References

[1] Aubert S., Barnes J.D., Abdeta C., Abi Nader P., Adeniyi A.F., Aguilar-Farias N., Andrade Tenesaca D.S., Bhawra J., Brazo-Sayavera J., Cardon G. (2018). Global Matrix 3.0 Physical Activity Report Card Grades for Children and Youth: Results and Analysis From 49 Countries. J. Phys. Act. Health.

[2] Church T.S., Thomas D.M., Tudor-Locke C., Katzmarzyk P.T., Earnest C.P., Rodarte R.Q., Martin C.K., Blair S.N., Bouchard C. (2011). Trends over 5 Decades in U.S. Occupation-Related Physical Activity and Their Associations with Obesity. PLoS ONE.

[3] Brownson R.C., Boehmer T.K. (2004). Patterns and Trends in Physical Activity, Occupation, Transportation, Land Use, and Sedentary Behaviors. Does the Built Environment Influence Physical Activity? Examining the Evidence.

[4] Healy G.N., Matthews C.E., Dunstan D.W., Winkler E.A.H., Owen N. (2011). Sedentary Time and Cardio-Metabolic Biomarkers in US Adults: NHANES 2003–06. Eur. Heart J..

[5] Dunstan D.W., Healy G.N., Sugiyama T., Owen N. (2010). ‘Too Much Sitting’ and Metabolic Risk—Has Modern Technology Caught Up with Us?. Eur. Endocrinol..

[6] Tremblay M.S., Colley R.C., Saunders T.J., Healy G.N., Owen N. (2010). Physiological and Health Implications of a Sedentary Lifestyle. Appl. Physiol. Nutr. Metab..

[7] Diaz K.M., Howard V.J., Hutto B., Colabianchi N., Vena J.E., Safford M.M., Blair S.N., Hooker S.P. (2017). Patterns of Sedentary Behavior and Mortality in U.S. Middle-Aged and Older Adults: A National Cohort Study. Ann. Intern. Med..

[8] Hege A., Lemke M.K., Apostolopoulos Y., Perko M., Sönmez S., Strack R. (2017). US Long-Haul Truck Driver Work Organization and the Association with Cardiometabolic Disease Risk. Arch. Environ. Occup. Health.

[9] Apostolopoulos Y., Lemke M., Sönmez S., Hege A. (2016). The Obesogenic Environment of Commercial Trucking: A Worksite Environmental Audit and Implications for Systems-Based Interventions. Am. J. Health Educ..

[10] Okunribido O.O., Magnusson M., Pope M.H. (2008). The Role of Whole Body Vibration, Posture and Manual Materials Handling as Risk Factors for Low Back Pain in Occupational Drivers. Ergonomics.

[11] European Commision Regulation (EC) No 561/2006 of the European Parliament and of the Council on the Harmonisation of Certain Social Legislation Relating to Road Transport and Amending Council Regulations (EEC) No 3821/85 and (EC) No 2135/98 and Repealing Council Regulation (EEC) No 3820/85 2006. https://eur-lex.europa.eu/eli/reg/2006/561/oj/eng.

[12] Gregory Smart Chair 2020. https://gregorychairs.com.au/ergonomics/smart/.

[13] SWINGSIT 2019. https://www.kickstarter.com/projects/1280484770/swingsit-best-active-sitting-chair-for-your-body.

[14] Routine Chair 2021. https://www.yankodesign.com/2021/12/27/this-smart-chair-morphs-position-physical-shape-as-your-posture-changes-through-the-day/.

[15] Amala Gracewin S., Chandru A., Kirubakaran D., Gomathi S. (2025). Smart Wheel Chair for Paralyzed Patients Using Advanced IoT. Proceedings of the 2025 International Conference on Intelligent and Innovative Technologies in Computing, Electrical and Electronics (IITCEE).

[16] Kumar A., Singh M., Singh M., Ramprabu J., Dubey A., Mujoo S. (2024). A Novel Electronic Wheel Chair Design Using Artificial Intelligence Assisted Smart Sensors and Controller. Proceedings of the 2024 5th International Conference on Intelligent Communication Technologies and Virtual Mobile Networks (ICICV).

[17] Esumi T., Takemura N. (2024). Posture Prediction in Response to Seat Angle Change with Smart Chair. Proceedings of the 2024 IEEE International Conference on Systems, Man, and Cybernetics (SMC).

[18] Lin B.-S., Liu K.-J., Tseng W.-H., Ahmed A.M., Wang H.-C., Lin B.-S. (2024). A Deep Learning–Based Chair System That Detects Sitting Posture. IEEE J. Biomed. Health Inform..

[19] Sigcha L., Pereira E., Lima A., Antunes J.T., Carvalhais D., Sousa D., Abreu A., Costa N., Cardoso P. (2023). A Noninvasive Smart Chair System for Monitoring Postures in Sedentary Workers. Proceedings of the 2023 IEEE 32nd International Symposium on Industrial Electronics (ISIE).

[20] Vibro Motor. https://www.jumia.com.ng/generic-dc-3v-60ma-9000-2000rpm-phone-coin-flat-vibrating-406236648.html#:~:text=Vibrating%20Vibration%2C%202%20leads,motor%20for%20cell%20phone.

[21] Pressure Sensor. https://www.vmanx.com/membrane-sensor/car-seat-occupancy-sensor-/2.html.

